# Digital twins to enable better precision and personalized dementia care

**DOI:** 10.1093/jamiaopen/ooac072

**Published:** 2022-08-18

**Authors:** Nilmini Wickramasinghe, Nalika Ulapane, Amir Andargoli, Chinedu Ossai, Nadeem Shuakat, Tuan Nguyen, John Zelcer

**Affiliations:** Swinburne University of Technology, School of Health Sciences, Department of Health and Bio Statistics, Hawthorn, Australia; Epworth HealthCare, Peter MacCallum Cancer Centre and Murdoch Children’s Research Institute, Australia; Swinburne University of Technology, School of Health Sciences, Department of Health and Bio Statistics, Hawthorn, Australia; Swinburne University of Technology, School of Health Sciences, Department of Health and Bio Statistics, Hawthorn, Australia; Swinburne University of Technology, School of Health Sciences, Department of Health and Bio Statistics, Hawthorn, Australia; Swinburne University of Technology, School of Health Sciences, Department of Health and Bio Statistics, Hawthorn, Australia; Swinburne University of Technology, School of Health Sciences, Department of Health and Bio Statistics, Hawthorn, Australia; Swinburne University of Technology, School of Health Sciences, Department of Health and Bio Statistics, Hawthorn, Australia

**Keywords:** digital twin, dementia, artificial intelligence, machine learning, clinical decision support

## Abstract

In this perspective paper, we want to highlight the potential benefits of incorporating digital twins to support better dementia care. In particular, we assert that, by doing so, it is possible to ensure greater precision regarding dementia care while simultaneously enhancing personalization. Digital twins have been used successfully in manufacturing to enable better prediction and tailoring of solutions to meet required needs, and thereby have enabled more effective and efficient deployment of resources. We develop a model for digital twin in the healthcare domain as a clinical decision support tool by extrapolating its current uses from the manufacturing domain. We illustrate the power of the developed model in the context of dementia. Given the rapid rise of chronic conditions and the pressures on healthcare delivery to provide high quality, cost-effective care anywhere and anytime, we assert that such an approach is consistent with a value-based healthcare philosophy and thus important as the numbers of people with dementia continues to grow exponentially and this pressing healthcare issue is yet to be optimally addressed. Further research and development in this rapidly evolving domain is a strategic priority for ensuring the delivery of superior dementia care.

## OBJECTIVE

The application of a digital twin, a digital replication of a whole or partial aspect of a physical entity, while having the ability to connect with the physical entity primarily through data transfer and simulate the physical entity’s activities in real-time,^1^ is revolutionizing many industries. Emerging from the aerospace and aviation sectors, more recently, the concept is being applied to genomics,^2^ aged care,^3^ and cancer care.^4^ Digital twins enable easy visualization, reasoning, experimenting, and forecasting of certain aspects of a physical world entity in a way that is efficient and cost effective.^5^ Given this, we proffer the application of digital twins to enable better precision and personalization for dementia care. Specifically, we note that, with the prevalence of dementia rising as a worldwide public health problem,^6^ the need for early intervention that is precise and yet personalized, timely, effective, efficient, efficacious, and of high value is paramount.

## BACKGROUND AND SIGNIFICANCE

Dementia is a worldwide public health problem, yet it is still not fully understood in terms of cause, prevention, diagnosis, and optimized management.^7^^,^^8^ It is estimated that the prevalence of dementia will triple from 50 million cases in 2018 to 152 million cases by 2050.^6^ Dementia is among the leading causes of disability and dependency in elderly people, with far-reaching implications for their quality of life as well as for their families and carers, for community, and for society.^9^^,^^10^ Brief provider–patient interactions, and the presence of often multiple symptoms and comorbidities in people with dementia, have contributed to missed, delayed, and even erroneous dementia diagnosis.^11–13^ Moreover, language barriers, illiteracy, chronic mental illness, hearing and visual loss, or a combination of these, further add to the difficulty in precise diagnosis and management.^14^ Furthermore, the best management options to slow the progression of dementia can vary depending on individual circumstances and risk factors.^15^ As such, precision, timeliness, effectiveness, and cost-effectiveness of planned management of dementia to achieve quality care and quality outcomes, using present-day methods and tools, is highly challenging and suboptimal.

To address this gap, we proffer the application of digital twin technology to be integrated with clinical practice in dementia diagnosis and care.

## MATERIALS AND METHODS

The utilization of a digital twin in the context of dementia care has the potential to serve as an intelligent decision support solution that can be used and accessed on a mobile device, tablet, or desktop computer to support clinicians, carers and family members of people with dementia.

**Figure 1.**
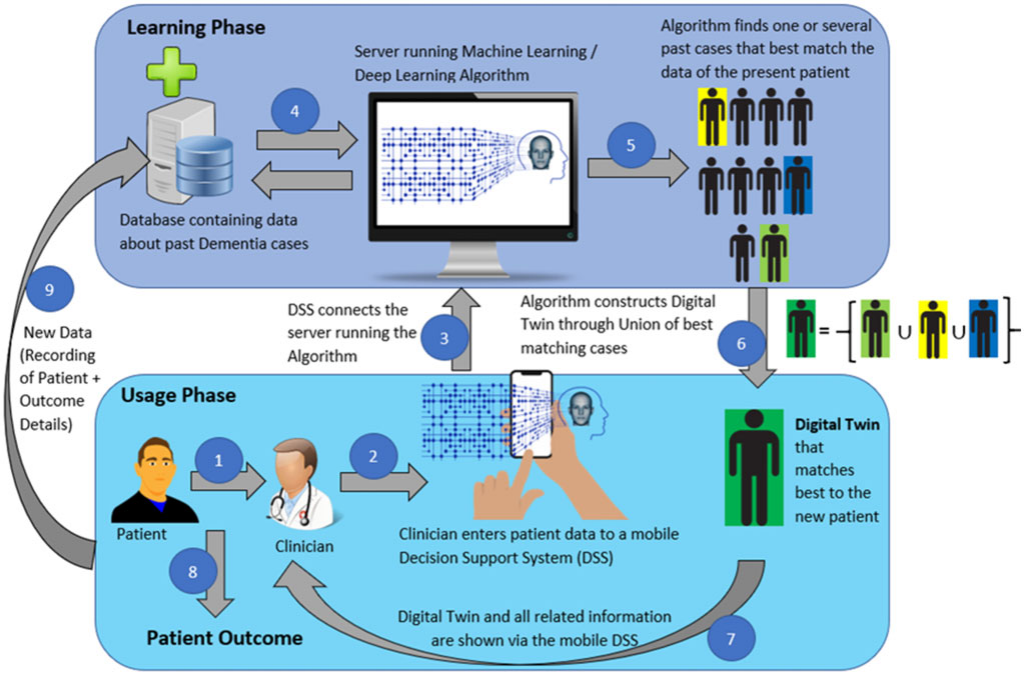
Proposed generic workflow for the use of Digital Twin by clinicians. Steps include: (1) Patient meets clinician; (2) Clinician enters patient data to a mobile Decision Support System (DSS); (3) DSS connects the server running the Machine Learning or Deep Learning Algorithm; (4) Algorithm connects to the database containing data about past dementia cases; (5) Algorithm finds one or several past cases that best match the data of the present patient; (6) Algorithm constructs Digital Twin through Union of best matching cases; (7) Digital Twin and all related information are shown to the end-user via the mobile DSS; (8) Clinician performs informed and precise diagnosis and treatment planning decisions; and (9) Details and outcomes about new patient get recorded as new data for future reference.

The process in use (Figure 1) would involve the end-user (clinician or carer) entering relevant data about a present patient into the Decision Support System (DSS). The data could include a patient’s dementia risk factors,^15^ current presentation, past or current treatments, progression of disease, and co-morbidities.

The DSS will securely connect to a remote server that runs a Machine Learning or a Deep Learning algorithm. This algorithm will, in turn, securely connect to a data repository containing rich data from an extensive cohort of dementia cases. The algorithm will identify one or several cases from the data repository that closely match the current patient’s data. The algorithm will then construct a single “model patient” through forming a union of the best matching cases identified from past data. This formed union will be the digital twin that most closely matches the current patient. In the event that a digital twin identical to the present patient cannot be formed, there will be of a quantitative similarity score that indicates the degree of similarity between the constructed digital twin and the current actual patient. In instances where a digital twin cannot be identified at all from the available past data, the algorithm should be able to detect such scenarios and advise the end user that a digital twin matching the entered data cannot be found.

## RESULTS

We identify 2 potential use cases for this digital twin framework: (1) Clinicians and (2) Carers and family members of people with dementia. Since the framework is mobile device enabled, this can be enhanced to provide value-added capabilities such as chatbots, call counseling, and telehealth consultations with clinicians.

In the clinician’s context, the clinician will use the digital twin to draw insight from past cases that are the same as, or similar to, the current patient to help inform diagnosis and management options. Thus, the clinician will be able to make better informed, and more precise, diagnosis and treatment planning decisions with the support of the additional information provided by the digital twin. Figure 1 depicts how this framework can be used by clinicians.

**Figure 2.**
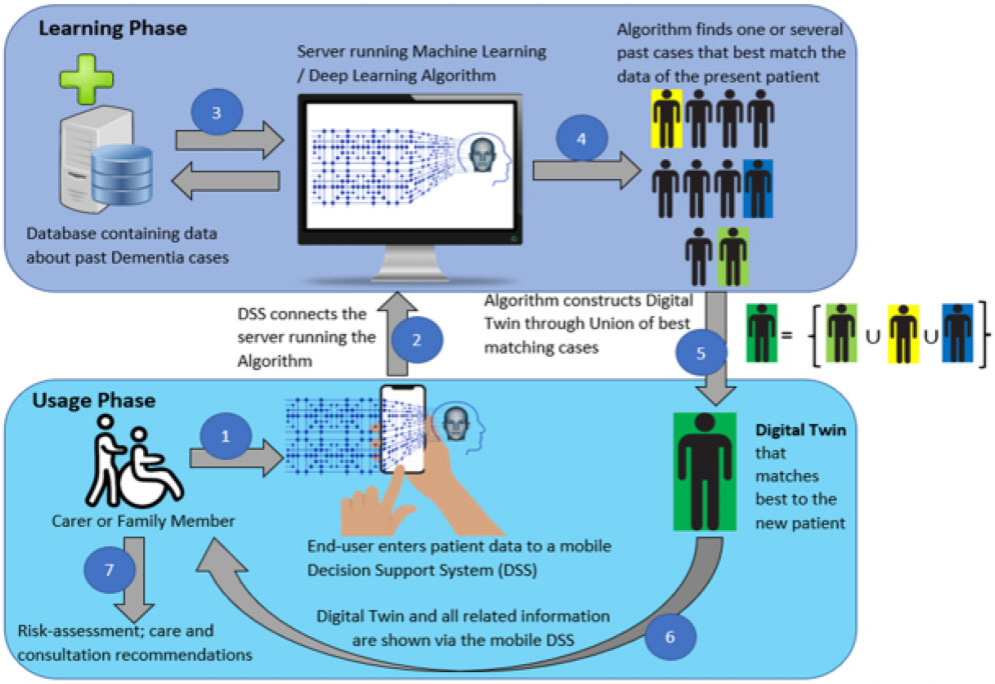
Proposed generic workflow for the use of Digital Twin by patients and carers. Steps include: (1) End-user enters patient data to a mobile Decision Support System (DSS); (2) DSS connects the server running the Machine Learning or Deep Learning Algorithm; (3) Algorithm connects to the database containing data about past dementia cases; (4) Algorithm finds one or several past cases that best match the data of the present patient; (5) Algorithm constructs Digital Twin through Union of best matching cases; (6) Digital Twin and all related information are shown to the end-user via the mobile DSS; and (7) Patient and/or Carer performs Self-Assessment, Risk-Assessment, or obtains evidence-based care and consultation recommendations.

For carers, including family members of people with dementia, the digital twin presentation may include risk-assessment, evidence-based care recommendations, and recommendations for further targeted clinical consultations. Figure 2 depicts a flowchart for how this framework can be used by carers.

## DISCUSSION

The key enablers for this digital twin framework are the rich and growing datasets available from large cohorts of dementia cases, mobile device-based technology support via the Internet of Things (IoT), and advances in Machine Learning and Deep Learning techniques and associated technologies. Rapid ongoing improvements will exponentially increase the potential and facility to design, develop, and implement such a framework.

Barriers include those that are generally applicable to Digital Health innovations and warrant consideration and further evaluation in the forward development and deployment of digital twins. These include complexity & performance issues; difficulty to validate; cost of development, implementation, and maintenance; data duality; lack of generalizability, expandability, and scalability; lack of streamlining with clinical workflow; privacy issues; surveillance capitalism; risks and accountability; policy and legislative challenges; slow or low adoption; personal biases; and competence with technology (or digital literacy).^16^

## CONCLUSION

Understanding dementia and providing better prevention, detection, treatment, and care is a significant challenge for clinicians, carers and people with dementia and is increasingly becoming a priority and key focus area for further understanding and optimization. Powerful and progressively sophisticated analytics and artificial intelligence tools, such as digital twins, offer the promise of precise, personalized, high value, high quality, effective, and efficient solutions for dementia prevention, prediction, diagnosis, and management. We note that this is also consistent with a value-based care approach where prudent deployment of limited resources to ensure maximum benefit is an important consideration.^17^ Thus, we urge that further research and development in this rapidly evolving domain is a strategic priority for ensuring the delivery of superior dementia care.

## FUNDING

This work was supported by funding received from the Barbara Dicker Brain Science Foundation in 2021.

## AUTHOR CONTRIBUTIONS

Substantial contributions to the conception or design of the work: NW. Substantial contributions to the acquisition, analysis, or interpretation of data for the work: NW, NU, AA, CO, NS, TN, and JZ. Drafting the work or revising it critically for important intellectual content: NW, AA, and JZ. Final approval of the version to be published: NW, AA, NU, CO, NS, TN, and JZ. Agreement to be accountable for all aspects of the work in ensuring that questions related to the accuracy or integrity of any part of the work are appropriately investigated and resolved: NW, AA, NU, CO, NS, TN, and JZ.

## CONFLICT OF INTEREST STATEMENT

None declared.
